# Association of Persistent Pulmonary Hypertension in Infants With the Timing and Type of Antidepressants In Utero

**DOI:** 10.1001/jamanetworkopen.2021.36639

**Published:** 2021-12-01

**Authors:** Trine Munk-Olsen, Veerle Bergink, Anna-Sophie Rommel, Natalie Momen, Xiaoqin Liu

**Affiliations:** 1 Department of Clinical Research, University of Southern Denmark, Odense, Denmark; 2 Department of Psychiatry, Icahn School of Medicine at Mount Sinai, New York, New York; 3 The National Centre for Register-based Research, Aarhus University, Aarhus Denmark

## Abstract

This population-based cohort study examines the risk of persistent pulmonary hypertension among newborns in Denmark after antidepressant exposure in utero.

## Introduction

Antidepressant exposure during pregnancy has been linked to increased risk of persistent pulmonary hypertension of the newborn (PPHN).^1^ Risks of PPHN have been explored for different types of selective serotonin reuptake inhibitors (SSRIs). However, risks after non-SSRI exposure and risks with timing of exposure in pregnancy across type of antidepressants have been insufficiently quantified or evidence remains conflicting.^2,3^ Given that PPHN is a potentially lethal complication, more detailed information on trimester- and antidepressant type–specific risk is essential.

## Methods

In this population-based cohort study, we identified 1 246 347 live-born singleton children born to 707 026 mothers from January 1, 1997, to December 31, 2016. Antidepressant use during pregnancy was ascertained from recorded prescriptions of antidepressants (anatomic therapeutic chemical [ATC] code N06A) dispensed on any date between 1 month before pregnancy and delivery.^4^ We investigated the association by timing of antidepressant exposure as follows: early pregnancy (defined as ≤20 weeks’ gestation) and late pregnancy (defined as >20 weeks’ gestation), and treatment was categorized into SSRI (ATC code N06AB) and non-SSRI (ATC code N06A excluding N06AB) antidepressants (Table 1). Information on race and ethnicity was not available for the present study. A newborn was categorized as having PPHN through a hospital contact with *International Statistical Classficiation of Diseases, Tenth Revision* codes of P29.3 or I27.0 within 7 days of birth identified from the patient registry.^5^ The Danish Data Protection agency gave permission for the study to be conducted, and no informed consent is required for register-based studies based on deidentified data. This study followed the Strengthening the Reporting of Observational Studies in Epidemiology (STROBE) reporting guideline.

**Table 1.** Classes of Antidepressant Treatment During Pregnancy

Antidepressant	ATC code	No. (%) of pregnancies^a^
SSRIs	N06AB	24 401 (2.0)
Citalopram	N06AB04	10 237 (0.8)
Sertraline	N06AB06	8053 (0.6)
Fluoxetine	N06AB03	5169 (0.4)
Escitalopram	N06AB10	1917 (0.2)
Paroxetine	N06AB05	1777 (0.1)
Fluvoxamine	N06AB08	<25 (<0.1)
Non-SSRIs^b^	N06AA, N06AF, N06AG, and N06AX	6875 (0.6)
Venlafaxine	N06AX16	3279 (0.3)
Mirtazapine	N06AX11	1404 (0.1)
Amitriptyline	N06AA09	898 (0.1)
Nortriptyline	N06AA10	799 (0.1)
Duloxetine	N06AX21	687 (0.1)
Mianserin	N06AX03	387 (<0.1)
Bupropion	N06AX12	202 (<0.1)
Clomipramine	N06AA04	170 (<0.1)
Tricyclic antidepressants^c^	N06AA02, N06AA07, N06AA11, N06AA12, N06AA16, N06AA17, N06AA21, N06AF01, N06AG02, N06AX06, N06AX18, N06AX22, and N06AX26	336 (<0.1)

Abbreviations: ATC, anatomic therapeutic chemical; SSRIs, selective serotonin reuptake inhibitors.

^a^ The sum of the number of pregnancies exposed to different types of antidepressants is more than 29 822 (the number of exposed pregnancies) because some women redeemed more than 1 type of antidepressant.

^b^ Non-SSRIs included SSRIs, tricyclic antidepressants, monoamine oxidase inhibitors, and tetracyclic antidepressants.

^c^ Tricyclic antidepressants included imipramine, lofepramine, protriptyline, doxepin, dosulepin, amoxapine, maprotiline, isocarboxazid, moclobemide, nefazodone, reboxetine, agomelatine, and vortioxetine.

Data were analyzed from March 1 to July 31, 2021. We estimated adjusted odds ratios (ORs) and absolute risk differences of PPHN by antidepressant exposure status using random-effects logistic regression with robust SE and mothers’ identification number as a cluster to account for the dependence between siblings. A detailed description of methods and potential confounders can be found in the eMethods in the Supplement. We also calculated the number of women who would need to be treated with antidepressants to cause 1 additional PPHN case as the inverse of the adjusted absolute risk reduction. A 2-sided significance threshold of *P* = .05 was used for the calculations. Data were analyzed in Stata, version 15.0 (StataCorp LLC).

## Results

Among the 1 246 347 live-born singleton children born to 707 026 mothers, the mean (SD) age of the mothers was 30.5 (4.9) years, and 44.9% were primiparous. Of 29 822 live-born singleton children born to mothers who used antidepressants, PPHN was identified among 79 children (2.6 per 1000 live births), in contrast to 1637 children (1.3 per 1000 live births) among the unexposed children (Table 2). The adjusted OR for PPHN after antidepressant exposure at any time during pregnancy was 1.29 (95% CI, 0.95-1.74). Of note, detailed results showed differences in risk by exposure early vs late in pregnancy; at 20 weeks’ gestation or less: OR, 0.80; 95% CI, 0.51-1.25; at more than 20 weeks’ gestation: OR, 2.01; 95% CI, 1.32-3.05. The absolute risk difference for the development of PPHN after exposure to antidepressants in late pregnancy was 1.3 per 1000 infants (95% CI, 0.2-2.4), suggesting that between 417 and 5000 women would need to be treated with antidepressants in late pregnancy to result in 1 additional PPHN case. Further, in late pregnancy, a more pronounced risk was observed with non-SSRI exposure (OR, 2.56; 95% CI, 1.54-4.25) than with SSRI exposure (OR, 1.36; 95% CI, 0.95-1.95) (*P* = .046).

**Table 2.** Adjusted ORs and Risk Differences of Persistent Pulmonary Hypertension by Antidepressant Exposure^a^

Antidepressant exposure during pregnancy	No.	Cases per 1000 infants	OR (95% CI)		Adjusted risk difference (95% CI) per 1000 infants^b^
			Crude	Adjusted^a^	
Exposure at any time during pregnancy					
No	1 216 525	1637 (1.3)	1 [Reference]	1 [Reference]	NA
Yes	29 822	79 (2.6)	1.99 (1.58 to 2.51)	1.29 (0.95 to 1.74)	0.4 (−0.1 to 0.9)
Timing of exposure, weeks’ gestation^c^					
≤20	27 832	73 (2.6)	0.99 (0.63 to 1.54)	0.80 (0.51 to 1.25)	−0.3 (−0.8 to 0.2)
>20	20 195	67 (3.3)	2.54 (1.59 to 4.05)	2.01 (1.32 to 3.05)	1.3 (0.2 to 2.4)
Type of antidepressants at any time during pregnancy^c^					
SSRIs	24 401	59 (2.4)	1.68 (1.28 to 2.21)	1.16 (0.83 to 1.61)	0.2 (−0.3 to 0.7)
Non-SSRIs	6875	25 (3.6)	2.40 (1.56 to 3.70)	1.55 (1.01 to 2.38)	0.7 (0 to 1.6)
Venlafaxine	2977	8 (2.7)	1.73 (0.85 to 3.49)	1.15 (0.56 to 2.36)	0.2 (−0.9 to 1.3)
Type of antidepressants exposure at >20 weeks’ gestation^c^					
SSRIs	17 272	48 (2.8)	1.90 (1.38 to 2.61)	1.36 (0.95 to 1.95)	0.5 (−0.1 to 1.0)
Non-SSRIs	3558	22 (6.2)	3.83 (2.34 to 6.24)	2.56 (1.54 to 4.25)	2.0 (0.4 to 3.6)
Venlafaxine	2024	7 (3.5)	2.24 (1.06 to 4.73)	1.45 (0.67 to 3.12)	0.6 (−0.9 to 2.1)

Abbreviations: NA, not applicable; ORs, odds ratios; SSRIs, selective serotonin reuptake inhibitors.

^a^ We identified potential confounders in our analyses using directed acyclic graphs.

^b^ Adjusted for maternal age at delivery, primiparity, maternal psychiatric history at delivery, maternal inpatient and outpatient treatment from 1 year before pregnancy to delivery, dispensing of antidepressants within 1 year before pregnancy, dispensing of other psychotropic prescriptions during pregnancy, dispensing of antiepileptic prescriptions during pregnancy, number of maternal nonpsychiatric hospital visits during pregnancy, smoking during pregnancy, marital status at delivery, highest education at delivery, and calendar year of delivery.

^c^ The number of subjects and cases add up to more than overall antidepressant-exposed children because women can use antidepressants at both periods and 2 different types of antidepressants. Defined groups of the timing of exposure and the types of antidepressants were mutually adjusted in the models.

## Discussion

The results of this population-based study suggest an association between antidepressant exposure in late pregnancy and increased risk of PPHN. However, we note that the absolute risk of PPHN was low and the number of mothers needed to be treated with antidepressants to result in 1 additional PPHN case varied substantially based on our analyses (from 417 to 5000 women). In contrast, there was no evidence of increased risk for exposure at or before 20 weeks’ gestation. Limitations of the study include potential exposure misclassification and a lack of power to study individual antidepressants. This study’s findings provide reassuring clinical information for a substantial proportion of women who stop or taper antidepressant use shortly after they become pregnant.

## Notes

Supplement. **eMethods.** Supplemental Methods
eReferences
