# Supplementary material for: Association of Persistent Pulmonary Hypertension in Infants With the Timing and Type of Antidepressants In Utero
Source: JAMA Netw Open. 2021 Dec 1;4(12):e2136639. doi: 10.1001/jamanetworkopen.2021.36639 (PMC8637253; doi:10.1001/jamanetworkopen.2021.36639)
Supplement: Supplement. — eMethods. Supplemental Methods eReferences [file jamanetwopen-e2136639-s001.pdf]

Supplemental Online Content

Munk-Olsen T, Bergink V, Rommel AS, Momen N, Liu X. Association of persistent pulmonary hypertension in infants with the timing and type of antidepressants in utero. *JAMA Netw Open*. 2021;4(12):e2136639. doi:10.1001/jamanetworkopen.2021.36639

eMethods. Supplemental Methods

eReferences

This supplemental material has been provided by the authors to give readers additional information about their work.

© 2021 Munk-Olsen T et al. *JAMA Network Open.*

eMethods. Supplemental Methods

Study population

We carried out a population-based cohort study using data from Danish national registers.[1] All live births and residents in Denmark are assigned a unique identification number in the Danish Civil Registration System,[2] which permits accurate linkage of individual-level data. We first identified 1,269,359 liveborn singletons born during 1997–2016 from the Danish National Medical Birth Registry.[3] We excluded 19,448 children who had missing or unrealistic gestational age or birth weight data (gestational age < 154 or > 315 days and birth weight < 300 or > 6,400 grams), and 3,564 children with chromosomal abnormalities (the International Classification of Diseases, 10th revision (ICD-10) codes: Q90–Q99). We included 1,246,347 children born to 707,026 mothers in the analyses.

Antidepressant exposure during pregnancy

Information on antidepressant use during pregnancy was retrieved from the Danish National Prescription Registry.[4] This register includes a record of all prescriptions dispensed in Denmark since 1995. It contains the anatomical therapeutic chemical (ATC) classification codes, the number of defined daily doses per package, the number of packages dispensed, and the dispensing date. The ATC code for selective serotonin reuptake inhibitors (SSRIs) was N06AB, and those for non-SSRI antidepressants were N06AA, N06AF, N06AG, and N06AX. The start of antidepressant use was indicated by the dispensing date of the first relevant prescription. We defined antidepressant use during pregnancy as at least one prescription dispensed on any date from one month before pregnancy until delivery. The start of pregnancy was ascertained from gestational age: Primarily based on the first- or second-trimester ultrasound scan; or, when no ultrasound data were available, the first day of the mother's last menstrual period.[3] We calculated the number of days exposed per prescription by multiplying the number of defined daily doses per package by the number of packages dispensed. We calculated the exact number of days exposed by adding the durations of all antidepressant prescriptions.

Outcomes of interest

Our primary outcome was persistent pulmonary hypertension of the neonate (PPHN), obtained from the Danish National Patient Registry.[5] The registry contains data on inpatients since 1977, and since 1995 outpatient and emergency visits are also included. PPHN was defined as the newborn having a hospital contact with ICD-10 codes of P29.3 or I27.0 within seven days of birth.

Potential confounders

We considered a broad range of potential confounders related to maternal characteristics in our analyses using directed acyclic graphs: psychiatric history at delivery, retrieved from the Danish Psychiatric Central Research Register[6] (ICD-8 codes 290–315; ICD-10 codes F00–F99); age at delivery (<25 years, 25–34 years, ≥35 years); primiparity (yes/no); inpatient and outpatient psychiatric treatment from one year before pregnancy until delivery (yes/no); dispensing of antidepressants within one year before pregnancy (yes/no), prescriptions for other psychotropic drugs (ATC codes N05 and N06 excluding N06A) during pregnancy (from one month prior to pregnancy until delivery; yes/no); prescriptions for antiepileptic drugs (ATC code N03) during pregnancy (yes/no); number of non-psychiatric hospital visits during pregnancy (0–1, 2–3, or ≥4); smoking during pregnancy (yes/no); marital status (married or cohabiting/single, divorced, or widowed); highest education (mandatory education/above mandatory education); and calendar year of delivery (1997–2000, 2001–05, 2006–10, or 2011–16). Data on these covariates came from the registers mentioned above as well as from Statistics Denmark's registers on socioeconomic status.[7]

Statistical analysis

Analyses were performed in Stata, version 16.0 (Stata Corp, College Station, TX). We used random-effects logistic regression to estimate the odds ratios (ORs),[8,9] absolute risk differences of PPHN, and their 95% confidence intervals (CIs), with robust standard error estimation and mother's identity as a cluster variable to account for the dependence between siblings., adjusting for the above-mentioned covariates. We also calculated the number of individuals who would need to be exposed to antidepressants during pregnancy to lead to each additional case of PPHN as the inverse of the adjusted absolute risk reduction.[10] Data were missing in 7.0% of the participants for one or more potential confounders; we applied 20 imputations using the Markov Chain Monte Carlo technique for imputing missing values.[11]

© 2021 Munk-Olsen T et al. *JAMA Network Open.*

To examine whether the associations between antidepressant exposure PPHN depending on the timing of exposure, we divided the exposure window into two groups based on the last menstrual period: ≤20 gestational weeks and >20 gestational weeks. We considered a child exposed to antidepressants in a specific time window if the dispensing date fell within the time window or if the number of days prescribed overlapped that time window. The timing of exposure was mutually adjusted for in the models. To study whether the associations varied with different types of antidepressants, we categorized antidepressant treatment into SSRIs and non-SSRIs. The types of antidepressants were mutually adjusted for in the models. We also examined the most common non-SSRI, venlafaxine use (ATC code N06AX16), specifically.

© 2021 Munk-Olsen T et al. *JAMA Network Open.*

eReferences

1. Thygesen LC, Daasnes C, Thaulow I, Brønnum-Hansen H. Introduction to Danish (nationwide) registers on health and social issues: structure, access, legislation, and archiving. *Scandinavian journal of public health.* 2011;39(7 Suppl):12-16.
2. Pedersen CB. The Danish Civil Registration System. *Scandinavian journal of public health.* 2011;39(7 Suppl):22-25.
3. Bliddal M, Broe A, Pottegård A, Olsen J, Langhoff-Roos J. The Danish Medical Birth Register. *European journal of epidemiology.* 2018;33(1):27-36.
4. Pottegård A, Schmidt SAJ, Wallach-Kildemoes H, Sørensen HT, Hallas J, Schmidt M. Data Resource Profile: The Danish National Prescription Registry. *International journal of epidemiology.* 2017;46(3):798-798f.
5. Schmidt M, Schmidt SA, Sandegaard JL, Ehrenstein V, Pedersen L, Sorensen HT. The Danish National Patient Registry: a review of content, data quality, and research potential. *Clinical epidemiology.* 2015;7:449-490.
6. Mors O, Perto GP, Mortensen PB. The Danish Psychiatric Central Research Register. *Scandinavian journal of public health.* 2011;39(7 Suppl):54-57.
7. Petersson F, Baadsgaard M, Thygesen LC. Danish registers on personal labour market affiliation. *Scandinavian journal of public health.* 2011;39(7 Suppl):95-98.
8. Neuhaus JM, Kalbfleisch JD. Between- and within-cluster covariate effects in the analysis of clustered data. *Biometrics.* 1998;54(2):638-645.
9. Begg MD, Parides MK. Separation of individual-level and cluster-level covariate effects in regression analysis of correlated data. *Statistics in medicine.* 2003;22(16):2591-2602.
10. Cook RJ, Sackett DL. The number needed to treat: a clinically useful measure of treatment effect. *BMJ (Clinical research ed).* 1995;310(6977):452-454.
11. Royston P, White IR. Multiple imputation by chained equations (MICE): Implementation in Stata. *J Stat Softw*. 2011; 45:1–20.

© 2021 Munk-Olsen T et al. *JAMA Network Open.*
